# Muscle Strength and Physical Performance in Relation to Dietary Intake Among Older Men and Women

**DOI:** 10.3390/nu18162684

**Published:** 2026-08-17

**Authors:** Małgorzata Kamila Pigłowska, Bartłomiej Konrad Sołtysik, Joanna Kostka, Tomasz Kostka, Agnieszka Guligowska

**Affiliations:** 1 Department of Geriatrics, Healthy Ageing Research Centre (HARC), Medical University of Lodz, Pomorska Street 247/249, 92-209 Lodz, Poland; 2 Department of Physioprophylaxis, Medical University of Lodz, pl. Hallera 1B, 90-647 Lodz, Poland

**Keywords:** dietary intake, muscle strength, physical performance, sarcopenia

## Abstract

Background: The aim of this study was to examine the relationships between dietary intake and two key components of sarcopenia in older, sex- and age-matched men and women: muscle strength and physical performance. Methods: The study included 620 carefully selected community-dwelling adults aged 60 years and more (310 men and 310 women) matched by sex and age (±1 year; median age: 66 years). Body mass and body mass index were assessed. Dietary intake was evaluated using 24 h dietary recall questionnaire and analyzed with Dieta 5.0 software. Handgrip strength (HGS) was measured using a hydraulic hand dynamometer, while physical performance using the Timed Up and Go test (TUG). Results: In men, but not in women, higher intakes of multiple dietary components, especially proteins, were associated with greater HGS. TUG was inversely related to animal protein, some amino acids, folate and vitamin C in men, whereas in women to copper, magnesium, vitamins C and E, riboflavin, and PUFA intakes. After multivariable adjustment, no nutrient remained associated with HGS in both sexes. In women, vitamin C intake remained inversely associated with TUG. Age was the strongest predictor of HGS, while age, physical activity (PA) and chronic diseases were significant factors associated with TUG in both sexes. Conclusions: Our findings show multifactorial nature of muscle physiology, suggesting that in preventive strategies adequate dietary intake integrated with PA and effective management of chronic diseases is required, as nutrition alone appears insufficient to preserve muscle function. The sex-stratified associations suggest that personalized dietary recommendations may be beneficial.

## 1. Introduction

According to European Working Group of Sarcopenia in Older People (EWGSOP), the three criteria of sarcopenia are: low muscle strength, low muscle mass and poor physical performance [1]. Muscle strength is recognized to be better than mass in predicting adverse outcomes and comes to the forefront in the sarcopenia algorithm [1]. Reduced muscle strength is also a key component of the frailty phenotype described by Fried et al. [2]. Progressive loss of muscle strength and physical performance influence functional status, independence in everyday activities, social participation and increase likelihood of adverse outcomes including falls, fractures, physical disability and mortality.

At present, appropriate nutrition and regular physical activity (PA) are the only evidence-based lifestyle interventions shown to be effective in the prevention and reversal of sarcopenia and frailty syndrome [3]. Changes occurring with aging cause decreasing diet quality, energy, and essential nutrients, and may lead to malnutrition [4] which is associated with muscle strength and physical performance [5]. The available literature indicates that certain dietary components have been proven to be effective in the prevention of sarcopenia [4]. However, existing studies present inconclusive results with regard to advancing age, sex, and particular elements of diet importance [6,7,8,9]. Therefore, we analyzed multiple parameters of dietary intake, expressed per kilogram of body mass, in a carefully selected group of older men and women matched for sex and age, and examined their relationships with muscle strength and physical performance.

## 2. Materials and Methods

The study was performed in 620 community-dwelling older subjects. Sex- and age-matched outpatients in the Geriatric Clinic of the Medical University of Lodz, Poland, volunteered to participate in extended examinations. The inclusion criteria were age of 60 years or more, domestic living environment, verbal logical contact to understand given questions and instructions, oral feeding, ability to walk (with or without auxiliary tools), signed informed consent for detailed assessment, completed dietary assessment, muscle strength examination, and completed Timed Up and Go test (TUG). Patients with dementia, those unable to walk or undergo muscle strength assessment and those receiving enteral nutrition were excluded from the study. The recruitment procedure was as follows: 310 men aged from 60 to 81 years old, being the outpatients of the Geriatrics Clinic who met the inclusion criteria, were enrolled to the study and then 310 sex- and age-matched (±1 year of age) women were subsequently included. All participants were of Caucasian ethnicity.

The study was approved by the Bioethics Committee of the Medical University of Łódź (project identification code: RNN/647/14/KB) and complies with the Declaration of Helsinki and Good Clinical Practice Guidelines.

### 2.1. Anthropometric Measurements

Anthropometric measurements were collected for all the participants. Body mass was measured with light clothes on and without shoes on a calibrated scale (Radwag, model WPT 100/200 OW, Poland) to the nearest 0.1 kilogram (kg) while body height was measured to the nearest 0.5 cm. Body mass index (BMI) was calculated [10].

### 2.2. Comorbidities and Number of Medications

Information on chronic conditions was obtained from patients’ medical histories. The assessed conditions included arterial hypertension, diabetes mellitus, myocardial infarction, chronic heart failure, stroke, chronic lung disease and osteoporosis. Information on the number of prescription medications currently taken by each participant was collected based on medical anamnesis. These data were used to characterize the study population and to assess the potential impact of chronic conditions and polypharmacy on the analyzed outcomes.

### 2.3. Dietary Assessment

Dietary intakes of foods and beverages consumed during the previous day were assessed using 24 h dietary recall questionnaires. All interviews were conducted by trained and experienced investigators. To improve the representativeness dietary recalls were collected only on days considered representative of the participants’ usual eating habits. Recalls were not obtained on days affected by study-related procedures or other circumstances that could temporarily alter dietary intake, such as fasting before clinical examinations. Given that older adults generally exhibit relatively stable and repetitive dietary patterns, day-to-day variation in dietary intake was expected to be lower than that observed in younger populations. Energy, water and nutrients intake were calculated using Dieta 5.0 software developed by the National Food and Nutrition Institute in Warsaw, Poland. Participants were asked to prepare a preliminary list of foods consumed on the assessed day prior to the interview. During the interview, detailed information was collected regarding food consumption, including the types of foods and beverages, portion sizes, preparation methods, additional ingredients, and time of consumption. To facilitate food visualization and improve the accuracy of portion size estimation, a photographic atlas of food products provided by the Polish Institute of Food and Nutrition was used [11].

### 2.4. Handgrip Strength

Upper extremities muscle strength was assessed with a hydraulic hand dynamometer (Saehan, Changwon-si, Republic of Korea) [12] according to a standardized protocol. Participants were instructed to stand upright with their arms at their sides and squeeze the dynamometer with maximal force. Three measures of each hand were performed with a 20–30 s pause between trials. The maximum value was used in the analysis, and the results were recorded to the nearest kilogram. Low handgrip strength was defined according to the EWGSOP recommendations (<16 kg for women and <27 kg for men) [1].

### 2.5. Timed Up and Go Test

The TUG was performed to evaluate the physical performance of the participants [13]. Patients were asked to rise from a seated position, walk three meters, turn around and return to a seated position. The time required to complete this test was measured and given in seconds. The two trials were done and the better result was taken. A score above 14 s in this test indicates an increased risk of falling.

### 2.6. Physical Activity

The Seven-Day Physical Activity Recall Questionnaire was used to assess the number of hours spent sleeping and engaging in physical activities during the previous 7 days (five weekdays and two weekend days). Physical activities were classified into four intensity categories: light, moderate, hard, and very hard. Weekly physical activity-related energy expenditure (PA-EE) was then calculated and expressed as kcal/kg/week [14,15].

### 2.7. Statistical Analysis

All analyses were conducted using Statistica software version 13.3 (Statsoft, Kraków, Poland). The normality of the data distribution was assessed using the Kolmogorov–Smirnov test. The results of the quantitative variables are presented as median and quartiles. To compare age, muscle strength, TUG performance, anthropometric indices, and dietary intake in both sex groups, Mann–Whitney test was used. The correlations between muscle strength, TUG and quantitative variables were assessed with Spearman correlation coefficients. Formal comparisons of sex-specific Spearman correlation coefficients between handgrip strength (HGS) or TUG performance and dietary variables were performed using Fisher’s r-to-z transformation. This approach was used to determine whether the strength of the observed correlations differed significantly between men and women.

Multivariable linear regression analyses were performed separately in women and men for each nutrient identified as significantly associated in the preliminary analyses to assess its independent association with HGS and TUG. Since both HGS and TUG values showed skewed distributions, logarithmic transformation was applied prior to the regression analyses. Three models were created for each dietary factor: Model 1 was adjusted for age only, while Model 2 was adjusted for age, the number of prescription medications, PA-EE, and chronic diseases that showed significant bivariate associations with handgrip strength and TUG performance in women and men, respectively. Given the strong predictive value of age, Model 3 was adjusted for the same covariates as Model 2, except that age was excluded to assess which factors remained significantly associated with HGS and TUG in the absence of this major determinant. The limit of significance was assumed to be a *p*-value of 0.05 or less for all analyses.

## 3. Results

The comparison of age, TUG, muscle strength, body mass, BMI, PA-EE, number of medications, chronic diseases, and dietary body mass-normalized intake of energy, water, lipids, carbohydrates, and fiber expressed per kilogram of body mass between women and men is presented in Table 1. Table 2 shows dietary intake of protein and amino acids and Table 3 shows minerals and vitamins expressed per kilogram of body mass in women and men. Women exhibited lower HGS and longer time required to complete TUG compared to men. According to the EWGSOP cut-off values for handgrip strength [1], 11 women (3.55%) and seven men (2.26%) were classified as having low muscle strength.

The comparison of dietary intake in women and men exhibited in absolute values is presented in Supplementary Tables S1–S3. Relative to current recommendations, both groups were characterized by inadequate intake of energy (below 30 kcal/kg of body mass) and protein (below 1.0 g/kg of body mass), according to European Society for Clinical Nutrition and Metabolism (ESPEN) recommendations [16]. Moreover, based on the 2024 Polish Dietary Reference Values for the Polish population, [17] both sexes presented low intakes of potassium (<3500 mg/day) and vitamin E (<8 mg/day for women and <10 mg/day for men), as well as excessive sodium intake (>1500 mg/day) relative to the Adequate Intake (AI), while the intake of calcium (<1200 mg/day), magnesium (<320 mg/day for women and <420 mg/day for men), copper (<0.9 mg/day), riboflavin (<1.1 mg/day for women and <1.3 mg/day for men), and vitamin C (<75 mg/day for women and <90 mg/day for men) was below the recommended dietary allowance (RDA).

Men were characterized with a higher intake of energy, water, total daily protein, carbohydrate and fat, mostly macro- and micro-nutrients, as well as vitamins and amino acids. However, when expressed relative to body mass, only sodium and starch intakes and calories-to-dietary-fiber ratio were higher in men. Women exhibited a higher intake of water, vitamin C, folate and manganese when expressed per kilogram of body mass (Table 1). Other components of dietary intake including all proteins and amino acids were similar in women and men.

Table 4, Table 5 and Table 6 present the relationships of HGS and TUG with anthropometric indices, PA-EE, number of medications, and dietary intake of energy, water, lipids, carbohydrates, and fiber (Table 4), protein and amino acids (Table 5), minerals and vitamins (Table 6) expressed per kg of body mass in women and men. Strong negative relationships were found between HGS and TUG results in both women and men. Older age was associated with lower HGS and longer TUG time, while higher body mass and BMI were significantly related to higher HGS in both sexes. PA-EE was positively related to HGS and negatively related to TUG in both women and men, whereas a higher number of medications was associated with lower HGS and a longer time required to complete TUG in both sexes.

In men, HGS was more strongly associated with many components of daily dietary intake as compared with women. Higher HGS was associated with higher intakes of energy, protein, including both animal and plant protein, and fat per kg body mass in men, whereas no such associations were found in women. Moreover, the higher muscle strength in men was related to body mass-adjusted higher intakes of all amino acids, phosphorus, magnesium, zinc, thiamine, niacin, vitamin B6, total saturated fatty acids, total monounsaturated fatty acids, total polyunsaturated fatty acids (PUFA), cholesterol, and starch. Higher calories to dietary fiber ratio was also related to higher HGS in men. In women, one association between HGS and dietary intake adjusted for body mass was identified—a negative association with water intake.

In both sexes, neither body mass nor BMI were associated with TUG performance. Intake of vitamin C adjusted for body mass was negatively correlated with TUG time (i.e., positively with better performance) in both sexes. Higher intakes of animal protein, some amino acids (lysine, histidine and tyrosine) and folate, were associated with shorter TUG times only in men. In women, copper, magnesium, vitamin E, riboflavin, and PUFA intakes, expressed per kg of body mass, were negatively associated with TUG time.

The relationships between HGS, TUG time, and dietary intake expressed in absolute values in women and men are presented in Supplementary Table S4.

Fisher’s r-to-z transformation test showed that the strength of several associations between dietary components and HGS differed significantly between women and men. Significant sex-related differences were observed for energy intake, total fat, saturated fatty acids, monounsaturated fatty acids, polyunsaturated fatty acids, carbohydrates, calories-to-dietary-fiber ratio, total protein, plant protein, most amino acids, starch, sodium, zinc, vitamin E, vitamin B6, thiamine and folate intake. None of the associations between dietary components and TUG performance showed significant differences between sexes.

Multivariable analysis of factors associated with HGS showed that, both in women and men, the previously observed associations between nutrients intake and HGS were no longer statistically significant in Model 1 after adjustment for age alone. Further adjustment in Model 2, including age, the number of medications, PA-EE, and chronic diseases, did not reveal any significant associations between nutrients intake and HGS. Age emerged as the strongest predictor of HGS and appeared to account for a substantial proportion of the observed relationship between nutritional factors and HGS. In Model 3, with age excluded, total protein, total fat, saturated fatty acids, monounsaturated fatty acids, starch, and calories-to-dietary-fiber ratio remained significantly associated with HGS in men, whereas for total energy intake, a trend toward an association was observed (*p* = 0.063). No significant dietary associations were found in women. In addition, the number of medications and PA-EE remained significant factors associated with HGS in both sexes.

Multivariable analysis of factors associated with TUG showed that Model 1, adjusted only for age, identified vitamin C intake as the only nutrient-related factor significantly associated with TUG performance in men. However, in Model 2, after adjustment for age, the number of medications, PA-EE, and chronic diseases, none of the dietary intake variables remained statistically significant. The association between vitamin C intake and TUG was attenuated and did not reach statistical significance (*p* = 0.067). In women, Model 1, adjusted only for age, showed that vitamin C intake and copper intake were significantly associated with TUG performance. After further adjustment in Model 2, only vitamin C intake remained to be the dietary factor significantly associated with TUG. In the fully adjusted model (Model 2), age, PA-EE, chronic heart failure and stroke were other significant factors associated with TUG in both men and women. In Model 3, vitamin C remained significantly associated with TUG in both men and women, together with PA-EE, chronic heart failure and stroke in men, and PA-EE, number of medications, chronic heart failure and stroke in women.

## 4. Discussion

In this study, multiple parameters of dietary intake were analyzed in a carefully selected cohort of older men and women matched for sex and age. Their associations with the two key components of sarcopenia, muscle strength and physical performance, were subsequently examined. The analysis focused primarily on dietary components expressed relative to body mass to better reflect actual intake across sex groups and to reduce confounding by body size, rather than on absolute intake values alone. This strategy may have minimized potential bias and improved the interpretability of the observed associations.

The use of body-mass normalized dietary variables seems particularly justified in older adults. In this population, substantial heterogeneity exists not only in nutritional status but also in body size and adiposity, meaning that comparable absolute intakes may correspond to different relative nutritional exposure. In addition, body mass-based metrics are already embedded in geriatric nutritional practice as recommendations for energy and protein intake, commonly expressed per kilogram of body mass [16,18].

In our study, men were characterized with higher intake of sodium and starch per kg of body mass, as well as a higher ratio of caloric-intake-to-dietary-fiber whereas women exhibited a higher intake of water, vitamin C, manganese and folate. These differences suggest that the male diet was closer to a typical Western dietary pattern, characterized by lower nutritional quality and higher sodium density, while the female diet was overall healthier and more consistent with healthy eating principles [19,20,21,22]. These findings are in line with previous studies showing that women consume fruits, vegetables, nuts, and seeds more often, whereas men choose foods rich in sodium and refined carbohydrate sources more frequently [19,20,21,22]. The higher vitamin C intake in women may also be considered in light of evidence indicating sex-specific differences in vitamin C metabolism and body mass-adjusted requirements [23,24]. A lower intake of vitamin C was required in women to achieve the same plasma concentration as in men [23]. Estimates of vitamin C requirements based on body weight suggest that an additional intake of approximately 10 mg/day is needed for every 10 kg increase in body mass [24].

Our results showed that higher body mass as well as BMI were related to higher muscle strength in men and women, which is consistent with the previous studies [25]. Elevated BMI in older people was shown to be associated with lower risk of malnutrition, lower muscle strength loss, lower functional decline and lower mortality risk [5]. The protective effect of higher BMI on weakness was also reported as a dose–response relationship revealing that in the underweight group, the risk of low HGS decreased with increasing BMI [25].

The important findings of our study indicate sex-related differences in the associations between muscle function and dietary intake in older women and men. In men, in contrast to women, HGS was associated with multiple components of daily dietary intake, including total energy intake. Higher energy consumption, along with increased intake of several other nutrients, were linked to greater HGS, suggesting a “higher consumption–stronger muscles” effect. When nutrients intake was adjusted for body mass, many remained statistically significant in men, indicating a relationship between diet and muscle strength.

Nutritional recommendations preventing muscle function loss consider both total calories intake and specific components of the diet [26]. A decline in total food intake is usually accompanied by a parallel decline in the intake of most nutrients [7]. The energy intake in older adults at the level of 30 kcal per kilogram of body mass is recommended, while in subjects with low BMI or those with chronic conditions it should be increased [16]. In our population, energy intake was approximately 21 kcal per kg body mass in both groups. The Israeli study showed that total energy intake was the only one factor associated with HGS despite overall diet being examined [6]. Moreover, the increase in energy intake of 100 kcal/day was associated with decreased prevalence of low HGS.

In our study, protein and amino acid intakes were positively associated with both muscle strength and physical performance in men, but not in women. However, although total- as well as animal- and plant-derived protein intake were associated with HGS, only animal protein intake was associated with physical performance. Protein is a key modifiable determinant of muscle metabolism, as it stimulates muscle protein synthesis and supports muscle integrity by providing essential amino acids (EAA) [26]. A concerning finding was the low protein intake in the study group, with median values of 0.89 and 0.87 g/kg body mass in women and men respectively, well below the recommended 1.0–1.2 g/kg body mass for older adults [16]. While most of the evidence presented protein intake and muscle relationship [4,8], inconsistent findings have also been reported [7]. In the Framingham Offspring cohort, the importance of animal-derived protein in maintaining muscle strength has been demonstrated showing that higher dietary intakes of total and animal protein, but not plant protein, were associated with a reduced rate of muscle strength decline in older men and women [9]. Apart from dietary protein intake, circulating amino acids are fundamental factors in controlling the rate of muscle protein synthesis and catabolism. EAAs, particularly leucine, isoleucine, and valine, stimulate this process via the mTORC1 pathway and may be particularly important in older adults with anabolic resistance [4,27]. Our results demonstrated some differences in the associations between protein and amino acid intake and muscle strength. All EAAs expressed per body mass were associated with HGS in men, but not in women. This pattern may reflect differences in amino acid kinetics, oxidative metabolism, hormonal regulation, and skeletal muscle characteristics. Compared with men, women exhibit a different muscle fiber type distribution, lower leucine oxidation rates, and higher basal muscle protein synthesis, which may attenuate their response to EAA supplementation [27].

Minerals may also contribute to the prevention of sarcopenia [4,28,29,30] through their roles in the regulation of inflammation and oxidative stress, particularly copper and magnesium, and through their involvement in protein synthesis, energy production, and muscle contraction, especially phosphorus and magnesium [31]. Lower intakes of magnesium, phosphorus, and selenium have been reported in sarcopenic individuals compared with non-sarcopenic controls [32]. Moreover, adequate dietary intakes of magnesium, calcium, phosphorus, copper, potassium, and vitamins B2, B9, C, and A were associated with a reduced risk of sarcopenia [28,30]. In this context, our sex-stratified analyses revealed distinct patterns of associations between mineral intake and muscle function. Specifically, higher phosphorus, zinc, and magnesium intakes were associated with greater muscle strength in men, while better physical performance was associated with higher copper and magnesium intake in women. These patterns may indicate sex-specific differences in the relevance of individual minerals to muscle function, although the underlying mechanisms remain to be established.

Antioxidants may help to preserve muscle mass and function in older adults by reducing age-related oxidative stress [33]. In our study, vitamin C intake was associated with muscle strength in men and physical performance in both men and women, while vitamin E intake was related to physical performance in women. Moreover, vitamin C was the only dietary factor that remained independently associated with physical performance in women after adjustment for age, PA-EE, chronic diseases, and number of medications. A similar trend was observed in men, although the association did not reach statistical significance (*p* = 0.067). These findings suggest that vitamin C may have a potential role in functional performance in older adults, particularly among women. Vitamin C is essential for collagen synthesis and maintenance of connective tissue, supporting muscle integrity and mobility [34]. The observed association was supported by experimental animal studies which showed that long-term vitamin C deficiency lead to skeletal muscle atrophy and impaired physical performance in both female and male mice [35]. These alterations were completely reversed following vitamin C supplementation, suggesting that adequate vitamin C availability is required for preserving muscle function [36]. Previous human studies have also reported beneficial effects of antioxidants on muscle strength and physical function, particularly in combination with exercise [33], as well as associations between higher intakes of vitamins A and C and a lower risk of sarcopenia [28].

Apart from antioxidants, B-group vitamins are important dietary elements for muscle strength and function in older adults, and their deficiency negatively affects the skeletal muscle function [29,37]. Vitamin B6 intake was associated with better chair rise test and greater HGS in healthy older European adults [38], while higher intake of vitamin B12, thiamin, riboflavin, and folate was associated with a lower risk of sarcopenia [28,32]. In our participants, B-group vitamins were also associated with physical performance (folate) and muscle strength (niacin, thiamine and vitamin B6) in men, while higher intake of riboflavin was related to better physical performance in both sexes.

In the present study, higher intake of PUFA was associated with higher muscle strength in men and better physical function in women. The Nurses’ Health Study with 30 years of follow-up showed that unsaturated fat intake, including PUFA, was related to better physical function [39]. These effects may be explained by the anti-inflammatory properties of PUFA and their role in muscle protein anabolism through activation of the mTOR signaling and reduction in insulin resistance [40,41]. Apart from PUFA, higher intake of saturated and monounsaturated fatty acids and cholesterol were related to higher muscle strength in men, most likely reflecting the co-occurrence of protein and fatty acids in animal-derived foods.

Despite the presence of several significant associations observed in the univariable correlation analyses, multivariable models did not confirm independent relationships between most dietary factors and muscle function. These findings highlight the complex and multifactorial nature of muscle physiology, suggesting that nutritional intake alone is unlikely to play a predominant role in determining functional outcomes. Among the analyzed factors, age emerged as a major determinant of muscle strength, where it accounted for a substantial proportion of the observed variability. Physical performance was primarily associated with age, PA-EE and the presence of chronic heart failure and stroke, emphasizing the importance of considering broader health-related and lifestyle factors when evaluating the relationship between nutrition and functional capacity. These findings are consistent with well-established evidence that aging is the primary driver of the progressive decline in muscle function and physical performance. Age-related changes in skeletal muscle, including reductions in muscle mass and quality, preferential loss of type II muscle fibers, impaired neuromuscular function, mitochondrial dysfunction, and chronic low-grade inflammation, contribute to the development of sarcopenia. At the same time, regular PA is recognized as one of the most effective strategies for preserving mobility and functional independence in older adults. Chronic heart failure and stroke further compromise physical performance through reduced exercise capacity, impaired neuromuscular function, and limitations in mobility, accelerating functional decline. Together, these findings emphasize that preventing muscle strength and physical performance loss in older adults requires an integrated approach combining adequate nutrition, regular PA, and optimal management of chronic diseases.

The present study has several limitations that should be acknowledged. First, its cross-sectional design precludes the establishment of causal relationships between dietary factors and muscle function. Second, the one-day dietary assessment may not fully reflect participants’ habitual long-term dietary intake. Another limitation was the lack of biochemical assessment of vitamin status, which restricted the ability to evaluate the participants’ actual nutritional status. Body mass normalization should be interpreted with caution because body composition was not available and the proportion of metabolically active tissue could not be considered. Despite these limitations, the study provides valuable insights into the associations between dietary factors and muscle function in older women and men.

## 5. Conclusions

The results of this study provide a generally consistent picture, suggesting that higher intakes of several dietary components are associated with better muscle strength and physical performance in older adults. Furthermore, our findings indicate that dietary factors, especially proteins, were more frequently associated with muscle strength in men than in women. However, after adjustment for relevant confounding factors, age emerged as the strongest determinant of handgrip strength in men, highlighting the predominant role of biological aging on muscle strength. Regarding physical performance, vitamin C intake was the only dietary factor that remained significantly associated with performance in multivariable analyses in women. Age, PA-EE, chronic heart failure and stroke were significant factors associated with TUG in both sexes. These results emphasize the multifactorial nature of muscle function loss, which is influenced by several nutritional, demographic, lifestyle, and health-related factors. Therefore, in preventive strategies, adequate dietary intake integrated with PA and effective management of chronic diseases is required, as nutrition alone appears insufficient to preserve muscle function. The sex-stratified associations suggest that personalized dietary recommendations may be beneficial.

## Figures and Tables

**Table 1:** Comparison of age, TUG, muscle strength, body mass, BMI, PA-EE, number of medications, chronic diseases, and dietary intake of energy, water, lipids, carbohydrates, and fiber expressed per kilogram of body mass between women and men.

Variable	Women N = 310	Men N = 310	*p*-Value
	Median (Q1; Q3)	Median (Q1; Q3)	
**Age (years)**	66.0 (63.0; 78.0)	66.0 (63.0; 78.0)	0.934
**TUG (s** **ec** **)**	7.11 (6.20; 8.25)	6.80 (5.78; 8.00)	**0.003**
**HGS (kg)**	25.0 (20.0; 30.0)	44.0 (36.0; 55.0)	**<0.001**
**Body mass (kg)**	68.2 (60.0; 78.5)	80.7 (72.5; 89.2)	**<0.001**
**BMI (kg/m^2^)**	27.8 (24.8; 31.3)	27.5 (25.0; 30.3)	0.172
**PA-EE (kcal/kg/week)**	281.5 (259.0; 318.2)	280.7 (254.0; 319.0)	0.761
**Number of medications**	5.0 (2.0; 8.0)	4.0 (1.0; 7.0)	0.073
DISEASES			
**Arterial hypertension, n (%)**	187 (60.3%)	193 (62.3%)	0.620
**Diabetes mellitus, n (%)**	43 (13.9%)	57 (18.4%)	0.126
**Myocardial infarction, n (%)**	16 (5.2%)	29 (9.4%)	**0.044**
**Chronic heart failure, n (%)**	85 (27.4%)	63 (20.3%)	**0.038**
**Stroke, n (%)**	20 (6.45%)	25 (8.06%)	0.439
**Chronic lung disease, n (%)**	40 (12.9%)	38 (12.3%)	0.809
**Osteoporosis, n (%)**	84 (27.1%)	21 (6.8%)	**<0.001**
DIETARY INTAKE			
**Energy (kcal/kg)**	20.9 (15.8; 27.2)	21.07 (15.8; 27.9)	0.447
**Water (m** **L** **/kg)**	28.6 (22.8; 37.3)	26.7(20.6; 34.1)	**0.004**
**Total fat (g/kg)**	0.70 (0.48; 0.95)	0.71 (0.50; 0.98)	0.343
**Total saturated fatty acids (g/kg)**	0.27 (0.18; 0.37)	0.27 (0.18; 0.38)	0.525
**Total monounsaturated fatty acids (g/kg)**	0.25 (0.16; 0.38)	0.27 (0.17; 0.39)	0.173
**Total polyunsaturated fatty acids (g/kg)**	0.09 (0.06; 0.15)	0.09 (0.06; 0.15)	0.615
**Long-chain polyunsaturated fatty acids (mg/kg)**	0.466 (0.060; 1.640)	0.455 (0.080; 1.710)	0.819
**Cholesterol (mg/kg)**	2.91 (1.86; 4.58)	2.97 (1.91; 4.54)	0.851
**Carbohydrates (g/kg)**	2.90 (2.10; 3.84)	2.85(2.10; 3.95)	0.597
**Sucrose (g/kg)**	0.47 (0.26; 0.77)	0.47 (0.23; 0.80)	0.851
**Lactose (g/kg)**	0.07 (0.03; 0.19)	0.08 (0.02; 0.19)	0.875
**Starch (g/kg)**	1.41 (1.00; 1.97)	1.57 (1.09; 2.20)	**0.023**
**Calories-to-dietary-fiber ratio (kcal/g)**	82.10 (65.32; 109.34)	92.76 (67.96; 123.65)	**0.005**
**Fiber (g/kg)**	0.24 (0.183; 0.34)	0.23 (0.17; 0.32)	0.088

BMI—body mass index; g—gram; HGS—handgrip strength; kcal—kilocalorie; kg—kilogram; mg—miligram; mL—milliliter; PA-EE—physical activity-related energy expenditure; *p*-value—value of statistical significance; Q1; Q3—Quartile 1; Quartile 3; sec—second; TUG—Timed Up and Go test.

**Table 2:** Comparison of dietary intake of protein and amino acids expressed per kilogram of body mass between women and men.

Variable	Women N = 310	Men N = 310	*p*-Value
	Median (Q1; Q3)	Median (Q1; Q3)	
**Total protein (g/kg)**	0.89 (0.64; 1.14)	0.87 (0.64; 1.13)	0.967
**Animal protein (g/kg)**	0.55 (0.38; 0.78)	0.57 (0.39; 0.77)	0.937
**Plant protein (g/kg)**	0.28 (0.22; 0.39)	0.29 (0.21; 0.39)	0.842
**Isoleucine (mg/kg)**	41.50 (30.02; 54.35)	40.87 (30.09; 53.51)	0.940
**Leucine (mg/kg)**	67.18 (47.76; 86.55)	65.58 (46.95; 86.24)	0.871
**Lysine (mg/kg)**	56.46 (41.32; 77.28)	57.49 (41.06; 74.99)	0.987
**Methionine (mg/kg)**	20.23 (14.80; 27.15)	20.38 (14.86; 27.02)	0.989
**Phenylalanine (mg/kg)**	38.25 (27.55; 50.28)	37.88 (27.33; 48.79)	0.972
**Threonine (mg/kg)**	35.08 (25.75; 46.69)	34.84 (26.27; 45.46)	0.970
**Tryptophan (mg/kg)**	10.79 (8.09; 14.62)	10.86 (8.13; 14.38)	0.907
**Valine (mg/kg)**	49.18 (36.00; 64.41)	48.76 (35.22; 64.29)	0.939
**Histidine (mg/kg)**	24.10 (17.25; 33.06)	24.33 (17.73; 31.67)	0.698
**Cystine (mg/kg)**	12.51 (9.51; 16.31)	12.60 (9.54; 17.03)	0.661
**Tyrosine (mg/kg)**	30.58 (22.05; 39.73)	30.88 (21.61; 39.62)	0.950
**Arginine (mg/kg)**	43.62 (33.14; 58.59)	44.22 (33.21; 55.67)	0.934
**Alanine (mg/kg)**	41.79 (30.30; 55.11)	41.94 (30.73; 53.13)	0.958
**Aspartic acid (mg/kg)**	78.74 (56.99; 103.20)	77.94 (56.82; 100.47)	0.901
**Glutamic acid (mg/kg)**	167.79 (122.14; 218.14)	166.05 (122.73; 220.91)	0.881
**Glycine (mg/kg)**	35.63 (26.29; 48.71)	36.69 (27.01; 49.01)	0.699
**Proline (mg/kg)**	59.84 (43.24; 77.79)	60.03 (42.67; 78.94)	0.902
**Serine (mg/kg)**	42.08 (31.32; 53.84)	41.15 (29.99; 53.22)	0.658

g—gram; kg—kilogram; mg—miligram; *p*-value—value of statistical significance; Q1; Q3—Quartile 1; Quartile 3.

**Table 3:** Comparison of dietary intake of minerals and vitamins expressed per kilogram of body mass between women and men.

Variable	Women N = 310	Men N = 310	*p*-Value
	Median (Q1; Q3)	Median (Q1; Q3)	
**Sodium (mg/kg)**	40.43 (29.78; 55.16)	44.16 (33.70; 56.77)	**0.012**
**Potassium (mg/kg)**	39.48 (28.667; 51.37)	37.01 (27.90; 49.50)	0.328
**Calcium (mg/kg)**	6.81 (4.14; 10.09)	6.19 (3.94; 9.28)	0.174
**Phosphorus (mg/kg)**	14.73 (10.62; 18.98)	13.98 (10.27; 18.38)	0.462
**Magnesium (mg/kg)**	3.68 (2.73; 4.81)	3.50 (2.65; 4.60)	0.237
**Iron (mg/kg)**	0.13 (0.10; 0.18)	0.12 (0.09; 0.17)	0.098
**Zinc (mg/kg)**	0.12 (0.09; 0.16)	0.12 (0.09; 0.15)	0.829
**Copper (mg/kg)**	0.014 (0.011; 0.020)	0.014 (0.009; 0.018)	0.061
**Manganese (mg/kg)**	0.065 (0.047; 0.095)	0.060 (0.044; 0.083)	**0.037**
**Iodine (mg/kg)**	1.71 (1.18; 2.49)	1.75 (1.17; 2.43)	0.844
**Vitamin C (mg/kg)**	1.01 (0.56; 1.75)	0.76 (0.43; 1.37)	**0.002**
**Vitamin A (μg/kg)**	10.87 (6.591; 17.68)	9.91(6.63; 15.43)	0.299
**Beta-carotene (μg/kg)**	37.63 (16.23; 65.87)	33.81 (14.98; 53.67)	0.122
**Vitamin E (mg/kg)**	0.09 (0.07; 0.13)	0.09 (0.06; 0.13)	0.710
**Thiamine (mg/kg)**	0.01 (0.01; 0.02)	0.01 (0.01; 0.02)	0.509
**Riboflavin (mg/kg)**	0.02 (0.01; 0.02)	0.02 (0.01; 0.02)	0.334
**Niacin (mg/kg)**	0.19 (0.14; 0.27)	0.21 (0.14; 0.29)	0.289
**Vitamin B6 (mg/kg)**	0.02 (0.02; 0.03)	0.02 (0.02; 0.03)	0.408
**Folate (μg/kg)**	3.11 (2.36; 4.20)	2.85 (2.14; 3.88)	**0.020**
**Vitamin B12 (μg/kg)**	0.03 (0.02; 0.06)	0.03 (0.02; 0.05)	0.766
**Vitamin D (μg/kg)**	0.03 (0.01; 0.04)	0.03 (0.01; 0.05)	0.278

kg—kilogram; mg—miligram; μg—microgram, *p*-value—value of statistical significance; Q1; Q3—Quartile 1; Quartile 3.

**Table 4:** Relationships between HGS and TUG and age, anthropometric indices, PA-EE, number of medications, and dietary intake of energy, water, lipids, carbohydrates, and fiber expressed per kilogram of body mass in women and men.

Variable	Women N = 310		Men N = 310	
	HGS	TUG	HGS	TUG
**Age**	−0.544 ***	0.481 ***	−0.649 ***	0.366 ***
**HGS**	-	−0.392 ***	-	−0.407 ***
**Body mass**	0.255 ***	−0.005	0.329 ***	−0.044
**BMI**	0.135 *	0.060	0.113 *	0.054
**PA-EE**	0.302 ***	−0.362 ***	0.281 ***	−0.360 ***
**Number of medications**	−0.255 ***	0.297 ***	−0.264 ***	0.250 ***
**Energy**	−0.086	−0.009	0.203 ***	−0.052
**Water**	−0.153 *	−0.042	0.004	−0.095
**Total fat**	−0.092	−0.027	0.220 ***	−0.073
**Total saturated fatty acids**	−0.110	0.045	0.193 **	−0.050
**Total monounsaturated fatty acids**	−0.088	−0.049	0.221 ***	−0.076
**Total polyunsaturated fatty acids**	−0.044	−0.140 *	0.182 **	−0.058
**Long-chain polyunsaturated fatty acids**	−0.036	−0.009	−0.029	−0.090
**Cholesterol**	0.013	−0.044	0.146 *	−0.078
**Carbohydrates**	−0.097	0.044	0.106	−0.009
**Sucrose**	0.024	−0.029	−0.080	0.090
**Lactose**	0.011	−0.104	−0.052	−0.058
**Starch**	−0.054	0.061	0.197 ***	0.014
**Calories-to-dietary fiber ratio**	−0.046	0.078	0.143 *	0.036
**Fiber**	−0.037	−0.078	0.053	−0.069

BMI—body mass index; HGS—handgrip strength; kg—kilogram; PA-EE—physical activity-related energy expenditure; TUG—Timed Up and Go test. * *p* < 0.05; ** *p* < 0.01; *** *p* < 0.001.

**Table 5:** Relationships between HGS and TUG and dietary intake of protein and amino acids expressed per kilogram of body mass in women and men.

Variable	Women N = 310		Men N = 310	
	HGS	TUG	HGS	TUG
**Total protein**	−0.011	−0.074	0.172 **	−0.100
**Animal protein**	0.011	−0.070	0.159 **	−0.127 *
**Plant protein**	−0.096	−0.052	0.134 *	0.007
**Isoleucine**	−0.001	−0.080	0.173 **	−0.110
**Leucine**	−0.008	−0.079	0.169 **	−0.106
**Lysine**	0.016	−0.073	0.158 **	−0.121 *
**Methionine**	0.013	−0.088	0.163 **	−0.102
**Phenylalanine**	−0.014	−0.073	0.185 **	−0.103
**Threonine**	0.005	−0.084	0.176 **	−0.112
**Tryptophan**	−0.007	−0.070	0.169 **	−0.103
**Valine**	0.000	−0.075	0.167 **	−0.107
**Histidine**	−0.004	−0.076	0.165 **	−0.129 *
**Cystine**	−0.022	−0.089	0.160 **	−0.068
**Tyrosine**	−0.018	−0.072	0.171 **	−0.115 *
**Arginine**	−0.025	−0.063	0.166 **	−0.084
**Alanine**	0.000	−0.066	0.152 *	−0.103
**Aspartic acid**	0.010	−0.067	0.160 **	−0.099
**Glutamic acid**	−0.029	−0.072	0.179 **	−0.103
**Glycine**	0.002	−0.062	0.170 **	−0.088
**Proline**	−0.039	−0.052	0.179 **	−0.086
**Serine**	−0.024	−0.082	0.166 **	−0.106

HGS—handgrip strength; TUG—Timed Up and Go test. * *p* < 0.05; ** *p* < 0.01.

**Table 6:** Relationships between HGS and TUG and dietary intake of minerals and vitamins expressed per kilogram of body mass in women and men.

Variable	Women N = 310		Men N = 310	
	HGS	TUG	HGS	TUG
**Sodium**	−0.099	0.022	0.100	0.001
**Potassium**	−0.008	−0.056	0.105	−0.053
**Calcium**	0.017	−0.109	0.066	−0.095
**Phosphorus**	0.003	−0.091	0.133 *	−0.095
**Magnesium**	−0.002	−0.114 *	0.112 *	−0.065
**Iron**	−0.023	−0.049	0.099	−0.033
**Zinc**	−0.012	−0.078	0.157 **	−0.079
**Copper**	0.043	−0.137 *	0.084	−0.040
**Manganese**	−0.085	−0.037	0.002	0.011
**Iodine**	−0.098	0.007	−0.053	0.043
**Vitamin C**	0.044	−0.183 **	0.082	−0.185 **
**Vitamin A**	−0.018	0.009	0.018	−0.085
**Beta-carotene**	0.019	−0.046	−0.029	−0.080
**Vitamin E**	−0.069	−0.128 *	0.093	−0.092
**Thiamine**	−0.006	−0.108	0.202 ***	−0.060
**Riboflavin**	0.002	−0.132 *	0.075	−0.107
**Niacin**	0.030	−0.042	0.172 **	−0.083
**Vitamin B6**	−0.019	−0.029	0.147 *	−0.103
**Folate**	−0.055	−0.101	0.111	−0.121 *
**Vitamin B12**	−0.064	−0.043	0.008	−0.070
**Vitamin D**	−0.078	−0.022	0.055	0.029

HGS—handgrip strength; TUG—Timed Up and Go test. * *p* < 0.05; ** *p* < 0.01; *** *p* < 0.001.

## Data Availability

The datasets used and analyzed during the current study are available from the corresponding authors on reasonable request.

## Supplementary Materials

The following supporting information can be downloaded at https://www.mdpi.com/article/10.3390/nu18162684/s1. Supplementary Table S1: Comparison of dietary intake of energy, water, lipids, carbohydrates, and fiber given in absolute values between women and men. Supplementary Table S2: Comparison of dietary intake of protein and amino acids given in absolute values between women and men. Supplementary Table S3: Comparison of dietary intake of minerals and vitamins given in absolute values between women and men. Supplementary Table S4: Relationships between HGS, TUG and dietary intake of energy, water, lipids, carbohydrates, and fiber given in absolute values in women and men. Supplementary Table S5: Relationships between HGS, TUG and dietary intake of protein and amino acids given in absolute values in women and men. Supplementary Table S6: Relationships between HGS and TUG and dietary intake of minerals and vitamins given in absolute values in women and men.

## Abbreviations

The following abbreviations are used in this manuscript:

AI	Adequate intake
BMI	Body mass index
EAA	Essential amino acid
EWGSOP	European Working Group of Sarcopenia in Older People
g	Gram
HGS	Handgrip strength
kcal	Kilocalorie
kg	Kilogram
mg	Miligram
mL	Milliliter
μg	Microgram
PA	Physical activity
PA-EE	Physical activity-related energy expenditure
RDA	Recommended dietary allowance
TUG	Timed Up and Go test
*p*-value	Value of statistical significance
Q1; Q3	Quartile 1; Quartile 3
sec	Second
