# Supplementary material for: Muscle Strength and Physical Performance in Relation to Dietary Intake Among Older Men and Women
Source: Nutrients. 2026 Aug 17;18(16):2684. doi: 10.3390/nu18162684 (PMC13516448; doi:10.3390/nu18162684)
Supplement: Supplementary file 1 [file nutrients-18-02684-s001.zip › nutrients-4458679-supplementary.pdf]

Supplementary Table S1. Comparison of dietary intake of energy, water, lipids, carbohydrates, and fiber given in absolute values between women and men.

Variable	Women N=310	Men N=310	p-value
	Median (Q1; Q3)	Median (Q1; Q3)	
**Energy (kcal)**	1434.34 (1112.1;1772.5)	1704.3 (1301.8;2223.9)	<0.001
**Water (ml)**	2011.9 (1576.1;2460.5)	2125.4 (1679.1;2631.0)	0.029
**Total fat (g)**	46.33 (34.11;64.32)	54.60 (40.51;81.04)	<0.001
**Total saturated fatty acids (g)**	17.87 (12.78;25.24)	21.53 (15.01;29.62)	0.000
**Total monounsaturated fatty acids (g)**	16.82 (11.73;25.02)	20.64 (14.53;30.63)	0.000
**Total polyunsaturated fatty acids (g)**	6.41 (4.39;9.49)	7.65 (5.02;11.98)	0.000
**Long-chain polyunsaturated fatty acids (g)**	0.03 (0.01;0.12)	0.04 (0.01;0.15)	0.292
**Cholesterol (mg)**	200.16 (126.98;319.57)	231.69 (156.23;357.53)	0.009
**Carbohydrates (g)**	196.95 (155.05;255.97)	233.31 (175.82;303.16)	<0.001
**Sucrose (g)**	31.75 (18.25;52.10)	38.27 (19.45;63.33)	0.039
**Lactose (g)**	5.33 (1.72;12.60)	6.65 (2.03;14.64)	0.094
**Starch (g)**	96.90 (73.94;129.79)	125.95 (91.67;168.74)	<0.001
**Fiber (g)**	17.14 (13.12;22.83)	18.17 (13.59;25.83)	0.017

g- gram; kcal – kilocalorie; mg – milligram; ml – millilitre; p-value - the value of statistical significance; Q1; Q3 – quartile1; quartile3.

Supplementary Table S2. Comparison of dietary intake of protein and amino acids given in absolute values between women and men.

Variable	Women N=310	Men N=310	p-value
	Median (Q1; Q3)	Median (Q1; Q3)	
**Total protein (g)**	59.5 (46.1;76.5)	71.2 (53.7;90.1)	<0.001
**Animal protein (g)**	38.4 (26.4;53.8)	45.0 (30.4;60.9)	<0.001
**Plant protein (g)**	19.9 (15.4;25.5)	23.8 (18.4;31.1)	<0.001
**Isoleucine (mg)**	2756.33 (2126.09;3660.56)	3285.72 (2479.86;4299.65)	<0.001
**Leucine (mg)**	4470.41 (3398.36;5816.48)	5237.77 (3852.43;6666.58)	<0.001
**Lysine (mg)**	3857.95 (2879.02;5209.20)	4582.07 (3370.21;5853.61)	<0.001
**Methionine (mg)**	1378.77 (1053.18;1833.36)	1625.36 (1200.51;2126.77)	<0.001
**Phenylalanine (mg)**	2599.63 (2000.65;3319.55)	3074.25 (2298.06;3912.58)	<0.001
**Threonine (mg)**	2394.75 (1850.42;3160.56)	2848.79 (2127.13;3586.58)	<0.001
**Tryptophan (mg)**	729.50 (581.26;975.83)	867.55 (658.10;1126.89)	<0.001
**Valine (mg)**	3305.16 (2557.28;4294.91)	3922.16 (2928.81;4958.00)	<0.001
**Histidine (mg)**	1595.16 (1232.78;2162.61)	1948.62 (1415.14;2582.57)	<0.001
**Cystine (mg)**	879.33 (657.73;1077.08)	1017.08 (769.66;1330.67)	<0.001
**Tyrosine (mg)**	2085.28 (1606.05;2695.22)	2470.44 (1770.30;3117.75)	<0.001
**Arginine (mg)**	2960.32 (2306.81;3889.98)	3549.94(2613.18;4533.12)	<0.001
**Alanine (mg)**	2850.71 (2139.44;3655.74)	3330.95 (2430.62;4313.52)	<0.001
**Aspartic acid (mg)**	5153.02 (4139.28;6822.85)	6205.79 (4574.19;7979.00)	<0.001
**Glutamic acid (mg)**	11644.20 (8859.32;14528.76)	13494.95 (10185.37;17289.22)	<0.001
**Glycine (mg)**	2439.99 (1823.79;3339.19)	2931.90 (2142.83;3879.23)	<0.001
**Proline (mg)**	4139.47 (3057.37;5340.77)	4859.65 (3528.43;6410.23)	<0.001
**Serine (mg)**	2876.32 (2168.01;3668.69)	3330.37 (2482.59;4200.62)	<0.001

g- gram; mg – milligram; p-value - the value of statistical significance; Q1; Q3 – quartile1; quartile3.

Supplementary Table S3. Comparison of dietary intake of minerals and vitamins given in absolute values between women and men.

Variable	Women N=310	Men N=310	p-value
	Median (Q1; Q3)	Median (Q1; Q3)	
**Sodium (mg)**	2803.48 (2175.818;3680.00)	3572.20 (2674.74;4554.68)	<0.001
**Potassium (mg)**	2661.86 (2046.15;3428.63)	2954.33 (2231.82; 3862.54)	<0.001
**Calcium (mg)**	453.94 (298.37;697.49)	497.90 (319.30;778.31)	0.077
**Phosphorus (mg)**	1006.40 (778.56;1280.75)	1126.85 (869.74;1464.76)	<0.001
**Magnesium (mg)**	261.57 (196.18;319.14)	282.70 (224.18;360.06)	<0.001
**Iron (mg)**	9.28 (7.35;11.66)	9.86 (7.75;13.28)	0.007
**Zinc (mg)**	8.15 (6.47;10.13)	9.33 (7.16;11.95)	0.000
**Copper (mg)**	0.97 (0.73;1.30)	1.06 (0.80;1.41)	0.021
**Manganese (mg)**	4.62 (3.51;6.00)	4.85 (3.68;6.32)	0.093
**Iodine (mg)**	122.24 (82.65;160.47)	139.07 (94.73;188.98)	0.000
**Vitamin C (mg)**	71.90(36.50;120.84)	60.86 (34.80;112.94)	0.290
**Vitamin A (µg)**	735.50 (469.59;1211.89)	804.48 (542.32;1232.71)	0.100
**Beta-carotene (µg)**	2499.66 (1176.72;4759.40)	2597.22 (1297.60;3992.22)	0.810
**Vitamin E (mg)**	6.39 (4.50;9.04)	7.44 (5.06;10.82)	0.002
**Thiamine (mg)**	0.93 (0.69;1.27)	1.08 (0.82;1.54)	0.000
**Riboflavin (mg)**	1.29 (0.98;1.67)	1.42 (1.08;1.87)	0.001
**Niacin (mg)**	13.37 (9.37;18.99)	16.25 (11.52;22.79)	0.000
**Vitamin B6 (mg)**	1.47 (1.09;1.99)	1.70 (1.22;2.25)	0.001
**Folate (µg)**	222.22 (168.43;271.78)	231.90 (175.80;305.39)	0.049
**Vitamin B12 (µg)**	2.30 (1.49;3.55)	2.74 (1.61;4.21)	0.017
**Vitamin D (µg)**	1.75 (0.97;2.94)	2.14 (1.13;3.78)	0.002

mg - milligram; µg - microgram; p-value - the value of statistical significance; Q1; Q3 - quartile1; quartile3.

Supplementary Table S4. Relationships between HGS and TUG and dietary intake of energy, water, lipids, carbohydrates, and fiber given in absolute values in women and men.

Variable	Women N=310		Men N=310	
	HGS	TUG	HGS	TUG
**Energy**	0.043	-0.001	0.367***	-0.070
**Water**	-0.047	-0.070	0.158**	-0.112*
**Total fat**	0.001	-0.038	0.357***	-0.106
**Total saturated fatty acids**	-0.018	0.040	0.308***	-0.080
**Total monounsaturated fatty acids**	-0.023	-0.054	0.343***	-0.099
**Total polyunsaturated fatty acids**	0.039	-0.150*	0.292***	-0.072
**Long-chain polyunsaturated fatty acids**	-0.023	-0.005	-0.004	-0.091
**Cholesterol**	0.079	-0.036	0.249***	-0.095
**Carbohydrates**	0.044	-0.021	0.246***	-0.009
**Sucrose**	0.085	-0.036	-0.011	0.086
**Lactose**	0.048	-0.102	-0.009	-0.068
**Starch**	-0.047	0.054	0.356***	-0.001
**Fiber**	0.106	-0.101	0.181**	-0.093

HGS - handgrip strength; TUG - Timed up and go test.
*p<0.05; **p<0.01; ***p<0.001

Supplementary Table S5. Relationships between HGS and TUG and dietary intake of protein and amino acids given in absolute values in women and men.

Variable	Women N=310		Men N=310	
	HGS	TUG	HGS	TUG
**Total protein**	0.109	-0.092	0.334***	-0.125*
**Animal protein**	0.100	-0.071	0.289***	-0.152*
**Plant protein**	0.049	-0.074	0.295***	-0.008
**Isoleucine**	0.111	-0.098	0.328***	-0.136*
**Leucine**	0.106	-0.096	0.322***	-0.133*
**Lysine**	0.110	-0.085	0.306***	-0.145*
**Methionine**	0.127*	-0.109	0.310***	-0.129*
**Phenylalanine**	0.113*	-0.093	0.347***	-0.129*
**Threonine**	0.120*	-0.100	0.335***	-0.137*
**Tryptophan**	0.109	-0.089	0.328***	-0.126*
**Valine**	0.117*	-0.096	0.322***	-0.131*
**Histidine**	0.102	-0.091	0.318***	-0.150*
**Cystine**	0.112*	-0.105	0.332***	-0.083
**Tyrosine**	0.098	-0.087	0.324***	-0.137*
**Arginine**	0.086	-0.079	0.341***	-0.112*
**Alanine**	0.110	-0.082	0.312***	-0.126*
**Aspartic acid**	0.130*	-0.092	0.318***	-0.119*
**Glutamic acid**	0.094	-0.083	0.334***	-0.121*
**Glycine**	0.102	-0.076	0.337***	-0.120*
**Proline**	0.075	-0.064	0.319***	-0.105
**Serine**	0.098	-0.098	0.320***	-0.129*

HGS - handgrip strength; TUG - Timed up and go test.
*p<0.05; **p<0.01; ***p<0.001

Supplementary Table S6. Relationships between HGS and TUG and dietary intake of minerals and vitamins given in absolute values in women and men.

Variable	Women N=310		Men N=310	
	HGS	TUG	HGS	TUG
**Sodium**	0.013	0.031	0.249***	-0.004
**Potassium**	0.119*	-0.075	0.264***	-0.085
**Calcium**	0.104	-0.129	0.160**	-0.109
**Phosphorus**	0.116*	-0.110	0.286***	-0.121*
**Magnesium**	0.124*	-0.135**	0.271***	-0.094
**Iron**	0.119*	-0.065	0.254***	-0.059
**Zinc**	0.118*	-0.107	0.352***	-0.110
**Copper**	0.174**	-0.157**	0.225***	-0.065
**Manganese**	0.010	-0.052	0.121*	0.003
**Iodine**	0.006	-0.004	0.061	0.056
**Vitamin C**	0.102	-0.184**	0.140*	-0.196**
**Vitamin A**	0.056	0.006	0.117*	-0.103
**Beta-carotene**	0.076	-0.058	0.049	-0.085
**Vitamin E**	0.023	-0.133*	0.200***	-0.104
**Thiamine**	0.105	-0.127*	0.344***	-0.087
**Riboflavin**	0.106	-0.131*	0.224***	-0.134*
**Niacin**	0.129*	-0.056	0.298***	-0.112*
**Vitamin B6**	0.094	-0.038	0.294***	-0.138*
**Folate**	0.090	-0.127*	0.256***	-0.144*
**Vitamin B12**	-0.012	-0.053	0.083	-0.087
**Vitamin D**	-0.027	-0.037	0.128*	0.022

HGS - handgrip strength; TUG - Timed up and go test.
*p<0.05; **p<0.01; ***p<0.001
